# Towards a Sustainable City for Cyclists: Promoting Safety through a Mobile Sensing Application

**DOI:** 10.3390/s21062116

**Published:** 2021-03-17

**Authors:** Pablo Boronat, Miguel Pérez-Francisco, Carlos T. Calafate, Juan-Carlos Cano

**Affiliations:** 1 Computer Languages and Systems Department, Universitat Jaume I (UJI), 12071 Castelló de la Plana, Spain; 2 Computer Science and Engineering Department, Universitat Jaume I (UJI), 12071 Castelló de la Plana, Spain; mperez@uji.es; 3 Computer Engineering Department (DISCA), Universitat Politècnica de València (UPV), 46022 Valencia, Spain; calafate@disca.upv.es (C.T.C.); jucano@disca.upv.es (J.-C.C.)

**Keywords:** traffic warnings, 4G, LTE transmission latencies, REST, location based services, road safety, sustainable mobility

## Abstract

Riding a bicycle is a great manner to contribute to the preservation of our ecosystem. Cycling helps to reduce air pollution and traffic congestion, and so, it is one of the simplest ways to lower the environmental footprint of people. However, the cohabitation of cars and vulnerable road users, such as bikes, scooters, or pedestrians, is prone to cause accidents with serious consequences. In this context, technological solutions are sought that enable the generation of alerts to prevent these accidents, thereby promoting a safer city for these road users, and a cleaner environment. Alert systems based on smartphones can alleviate these situations since nearly all people carry such a device while traveling. In this work, we test the suitability of a smartphone based alert system, determining the most adequate communications architecture. Two protocols have been designed to send position and alert messages to/from a centralized server over 4G cellular networks. One of the protocols is implemented using a REST architecture on top of the HTTP protocol, and the other one is implemented over the UDP protocol. We show that the proposed alarm system is feasible regarding communication response time, and we conclude that the application should be implemented over the UDP protocol, as response times are about three times better than for the REST implementation. We tested the applications in real deployments, finding that drivers are warned of the presence of bicycles when closer than 150 m, having enough time to pay attention to the situation and drive more carefully to avoid a collision.

## 1. Introduction

Currently, local transport habits still have serious consequences both on ecology and health. The model provided in [1] proposes that the use of cars would require 97% of forested land to absorb their CO_2_ emissions; their example scenario is for Palermo Province in Italy, but it provides an idea of the impact of local transport. Furthermore, the impact on health has been studied for years. References [2,3] proved, respectively, the effects of traffic pollution on lung cancer and on the risk for stroke. Cities are now restricting diesel motorized vehicles in town centers.

The use of alternative vehicles such as bicycles or scooters is increasing all over the world at the same time that our environmental awareness is growing. Using bicycles is one of the most effective changes in our common daily commuting to reduce our pollution impact on the planet. However, the coexistence of bicycles with heavy vehicles is a main problem due to the differences in terms of speed, visibility, and vulnerability. In the European Union, twenty-two-thousand eight-hundred fatalities were estimated in 2019. Forty-seven percent of these fatalities correspond to vulnerable road users (VRUs), increasing up to 70% in urban areas [4].

To help this situation, an alarm system to prevent drivers and VRUs from accidents is an essential requirement. Our system would alert drivers when VRUs, such as bikes or electric scooters, are close, anticipating a possible collision. Drivers should have enough time to reduce their speed and to pay more attention.

Traffic warning systems could be implemented by detecting vehicles directly along with direct communication between them or by regularly sending the position and other motion data to a centralized server. The server tracks the vehicles’ trajectory, and it sends alarms to the concerned vehicles when needed.

The two approaches have benefits and drawbacks, and they could even be complementary [5]. However, the more inexpensive and easy solution to adopt is to use conventional smartphones. If phones are the only sensing devices used, direct communication between vehicles (using WiFi or Bluetooth) does not provide enough range for our target goal, and in addition, connection establishment is too slow, failing to provide on-time alerts [6].

We developed an Android application for smartphones and, also, for the time being, a simplified version of the server for testing. The application, running on smartphones, regularly sends the position of the vehicle to the server, getting back traffic alarms from the server, alerting about dangerous situations. For instance, a car can be alerted when bikes are closer than a given distance. A graphical interface based on OpenStreetMap can be optionally shown with the current position. We call this application SafeCyclists.

Two application layer protocols were designed and developed to interact with the server. The protocols differ on the level of the communications stack in which they were implemented. One of the protocols, which we call SafeCyclistsHTTP, uses an APIRest where a TCP connection is established when interacting with the server. Concerning the other protocol, named SafeCyclistsUDP, all interactions with the server are made with independent UDP messages, thus without establishing a connection at the transport communications layer.

The goal of this preliminary work is twofold. First, we wanted to check the performance differences between the two implemented protocols. Then, we wanted to prove the feasibility and limits of the warning system by testing it in real scenarios.

The rest of the paper is organized as follows: the next section provides an overview of related works on this topic. Then, in Section 3, we detail the proposed system architecture. Section 4 describes the proposed Android application that was developed. Afterward, experimental results are presented in Section 5 and a validation of the correct functionality of the system is provided in Section 6. Finally, in Section 7, we present the main conclusions of this work and refer to future works.

## 2. Related Work

In the literature, we can find different contributions addressing traffic safety and autonomous or assisted driving. Some high-end cars include collision detection systems to warn drivers and to automatically stop the vehicle. However, our goal is to validate a non-critical alert sensing system based on hardware that most drivers and cyclists already have with them: smartphones.

Reference [7] is an example of instrumentation for vulnerable road users. The authors stated that few works have been done to increase the safety of VRUs, where they are an active part of an intelligent cooperative system. As a first step, they explored an experimental prototype of instrumentation system for pedestrians. Furthermore, in [8], the authors provided a study of bicycle kinematics to detect the behavior in dangerous situations. Our work goes one step forward, being based on the ubiquity of smartphones, as they already pointed out, to maximize user adoption.

Works such as [6,9] proposed an Android application to send GPS data to a server, which then sends alerts to drivers. Our present work is probably complementary to theirs as we focus on the communication times to validate such an alarm system. We compare high and low level communication architectures in realistic scenarios under different conditions to determine the most suitable solution. Another related work is [10], which was based on IEEE 802.11p, and so, it could not be easily adopted.

In [11], a solution was proposed for intersections between motorized vehicles that cross bike lanes in turning maneuvers. They proposed an algorithm to prevent accidents in these situations, sending alerts to connected vehicles. However, they did not focus on the technological part of the alarm system.

Reference [12] provided the results of an experimental alarm system for car-bike cohabitation. A deep study on the accuracy of the smartphone’s GPS was presented, concluding that there is an error lower than 5 m in most cases. Furthermore, they measured the communications latency, which was found to be about 0.646 s using 3G cellular phone systems, but there was no hint about the communications implementation. Their conclusion was that such a traffic alarm system is feasible.

In the work presented in [13], the authors defended the idea that the best way to prevent accidents with vulnerable road users is a collaborative communications system, while specialized sensors are almost limited to line of sight conditions. They modeled and simulated the usage of low range 802.11p communications in a particular type of street intersection. Their results pointed out how difficult it is to generate critical safety warnings.

A complete research review about instrumented bikes to study transportation behavior, safety, and maintenance can be found in [14]. This review did not include studies such as ours because merely carrying a smartphone is not considered as an instrumented bike. However, the authors confirmed that one of the aspects needing more attention is using instrumented bikes, allowing informing other road users about position and speed to improve safety.

One of the first works around instrumented bikes was [15]. In this project, bicycles were instrumented to collect data about the cyclist and the environment, making the bike a mobile sensor network. The collected data were sent to a server, accessible to users. In this case, the goal was to check data about the trip and the health of bikers, and not about traffic safety.

Other examples of instrumented bikes to improve cyclists’ safety can be found in [16,17]. These two works proposed bicycles rear tracking systems. In [17], an experimental laser sensor, to be installed on bikes, was presented. It detects and tracks vehicles behind the bike. The system is complex, and it needs extra equipment, including a battery. This makes it difficult for by many cyclists to adopt it. Even if a dangerous situation is detected, drivers have to be alerted with lights or sounds, and the range of the system (25 m) makes it operational only in urban scenarios as, if the relative velocity between vehicles is greater than 11.9 m/s (42.84 km/h), cars cannot stop before the collision. In [16], the detection was done with a video camera connected to an on-board computer with computer vision techniques to detect rear-approaching vehicles.

Car manufacturers, led by Volvo [18], have developed bike detection systems to assist drivers, which is an interesting improvement for traffic safety. The Volvo system is based on shape image detection. Images are taken from a front camera. Only typical cyclist shapes traveling in front of the car, and moving in the same direction, are detected, and only with adequate light conditions. Furthermore, Volvo cars, in collaboration with POC and Ericsson, presented a project similar to ours in 2015 [19]. However, the project seems to have been abandoned, and to the best of our knowledge, no data about their results have been published.

Psychological or social aspects are also relevant for road safety applications. Despite this being a topic that is outside the scope of this paper, it would be interesting to know the level at which an application such as ours will be adopted by users. Furthermore, given that technology (e.g., smartphones, headphones, navigators) is one of the most important sources of distraction for road users [20,21,22], we should evaluate the convenience of road safety applications. In any case, we took this drawback into account as the application can be used with the phone’s screen turned off, and users are warned of danger through an acoustic alarm.

In this paper, we provide a detailed analysis of the communications architecture, given that it is critical for the alarm system. A considerable amount of work has been devoted to the communication of vehicles, both among them or with an external infrastructure. A recent revision can be found in [5]. The authors proposed to develop an adaptive general communication framework, which would take into account communication conditions and the quality of services required by the applications. In that case, the alarm system we study would be one of these applications. Both in [5] and in [23], it was exposed that aspects such as road safety and, in general, the more ambitious goal of autonomous driving will need flexible and adaptive approaches involving all communication technologies available.

## 3. System Architecture

**Figure 1:**
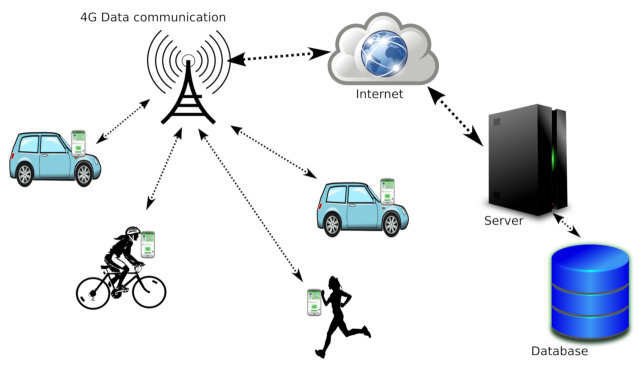
System architecture. Vehicles are connected to the cloud server through smartphones and the 4G cellular infrastructure.

The architecture of our proposed solution is composed by vehicles carrying smartphones, the 4G LTE cellular networks, and a centralized cloud server accessible via the Internet, as shown in Figure 1.

Phones periodically send their position to the server (at specific time periods or when a distance threshold is surpassed). The server will continuously update each phone’s position in the database.

If configured by the users, when phones send their position, they request alerts from the server. Different alerts can be chosen. The server checks if the phone is an appropriate target for the different demanded alerts and provides a reply. Finally, if any alert is received by the phone, a warning is shown to the user, and optionally, a sound is played back until the server updates its status to indicate that the alert conditions no longer apply.

In this work, our focus is on the interactions between the clients and server. The goal is to verify if, with conventional phones and standard data communication services as offered by mobile phone companies, an alert system such as the one described is feasible. We evaluated our system to test its limits under typical signal levels according to the 4G LTE coverage available in many countries.

### Time Constraints and Warning Distance

Assuming that a vehicle should have the possibility to stop before a collision, the distance at which a proximity warning is triggered depends on three factors: the response time of the system, the reaction time of the driver, and the distance needed to stop the vehicle. Let us analyze these factors from a conservative point of view, despite hat they can be further tuned later on, or even dynamically adapted to variable conditions.

In our analysis, we consider a quite extreme case where a car is moving at 80 km/h (the legal limit for secondary roads in many countries, 90 km/h being the legal limit for trunk roads; VRUs are not allowed on highways) in the opposite direction of a bike moving at 20 km/h; thus, the relative approximation speed is 100 km/h. This is reasonable for most road scenarios, and it should be an upper limit for urban scenarios.

It is widely accepted that the reaction time of a driver ranges between 1 and 2 s. In [24], a value of 1.9 s was said to represent the 85th percentile for the general population. Hence, the distance traveled by a car at 80 km/h during such a time period is 42.2 m.

Although the braking distance depends on many factors in addition to the speed (weight of the car, braking system, road conditions, etc.), we consider that stopping a car at 80 km/h requires about 40 m [25].

**Figure 2:**
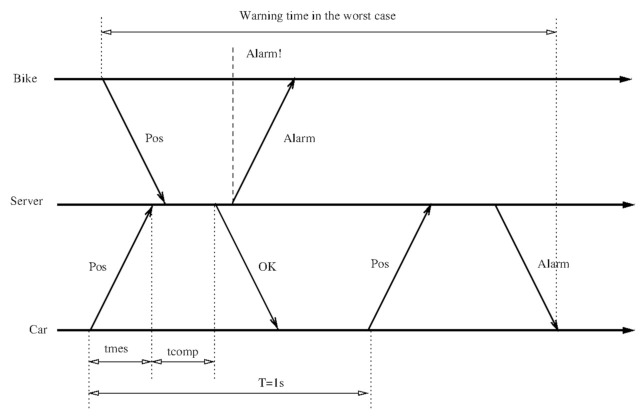
Worst case situation to receive a proximity warning: the server receives the position update from the bike just when it has started to process the car position update looking for alarms. It is possible that the recent message from the bicycle is not taken into account, causing the alarm to be delayed until the next message is processed.

Finally, we have to consider the impact of the system response time itself. That is, we need to evaluate the worst case, i.e., the maximum time between a message with a position triggering an alarm and the time when that alarm message arrives at the vehicles. As shown in Figure 2, the worst case happens when the message of one of the vehicles arrives just after the message of another close-by vehicle (while the server is looking for its alarm state). This time is the position sending period (1 s) plus the response time (including sending the position, the server’s computing time, and the response from the server). Experimentally, we obtained response times of about 0.1 s, but the application waits for the answer by up to 1 s. We can conclude a worse case of 2 s. Consequently, we assume that, during 2 s, the vehicles are approaching at 100 km/h, which results in a traveled distance of 55.5 m.

In Figure 2, tmes represents the communication time for messages (to/from the vehicle), and tcom is the server’s computation time; such a time involves updating the database and checking if the sender vehicle has proximity alerts. *T* is the position sending period (1 s).

The sum of the three distances results, from a quite conservative point of view, in a distance threshold to issue warnings of 42.2 + 40 + 55.5 = 137.7 m.

## 4. The SafeCyclists Android Application

**Figure 3:**
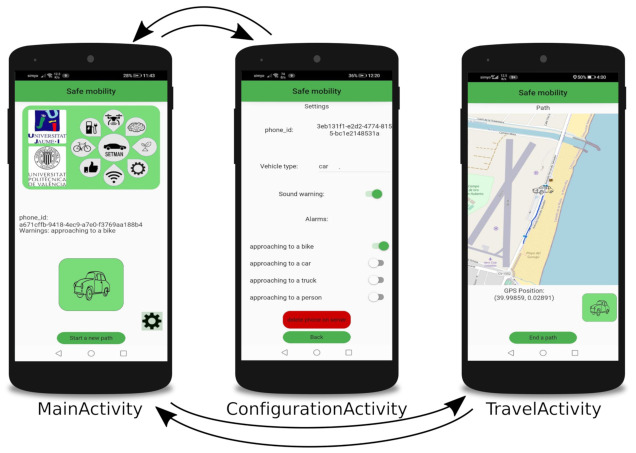
Available activities for the developed Android application.

The Android application we developed for our tests, SafeCyclists, has three activities. MainActivity is presented when launching the application. The first time that the application is executed, the user can only register the phone in the server anonymously. In the following executions, the user can switch to the other two activities, ConfigurationActivity and TravelActivity. Figure 3 shows the activities and how the user can alternate them.

In ConfigurationActivity, users can choose the type of vehicle, subscribe to receive different alarms (at the time being, only the proximity alarm is implemented), enable or disable the audio alarm, and delete their registration from the server.

TravelActiviy represents the main use of the application. Every second, the phone sends its position and listens for alarms. If an alarm is notified by the server, then a visual alert is shown, and an audible alert is produced. The phone will show the alert until the server answer does not include any alert notification. Furthermore, the current position and the trajectory are shown by using OpenStreetMaps.

In this work, we focus on the communications part, detailing the interaction between the application and the server. Two communication protocols were designed and implemented to test the impact on the response time; they were based, respectively, on the HTTP and the UDP protocols, and we call these variants SafeCyclistsHTTP and SafeCyclistsUDP. Below, we provide a brief description of both communication protocols.

Concerning the SafeCyclistsHTTP protocol, the communication with the server is implemented using a REST API where the application establishes a TCP connection with the server for each interaction. For instance, TravelActivity sends the server an HTTP request with the smartphone position every second, and an answer with the state of the alarms is received.

Responses that arrive later than a second are marked as outdated for the purposes of our study, and they are ignored by the phone, although the server may have used that information to update the position of the phone. In the case of connection errors, we consider the request as lost.

Regarding the SafeCyclistsUDP protocol, the interactions with the server are made using independent messages. The messages are programmed directly over the UDP transport protocol, and so, no connection is explicitly established. The position and alarm subscriptions are sent in one UDP message, and the answer from the server is received by a different thread with a timeout of one second (the period between requests). After one second waiting for the answer from the server, the thread continues, and the message is marked as outdated. As in the previous case, the phone cannot determine if its position has been updated in the server

## 5. Experimental Results

In this work, we focus on the system response time, which is the critical part of our solution, to determine its feasibility. The results are divided in terms of the type and goal of the experiment. First, we compare the two proposed protocols, and we assess the response time in different contexts and by varying the speed of the vehicles. Then, we perform additional tests to check the influence of other factors such as the radio signal power, the number of simultaneous users, or the battery consumption.

### 5.1. Comparing the Response Time of the UDP and HTTP Versions

As has been exposed, two versions of the communications protocol were implemented. One uses a REST API over HTTP, which is easier to develop; the other was programmed directly over the UDP protocol. We wanted to verify the impact of these technologies on the response time.

**Figure 4:**
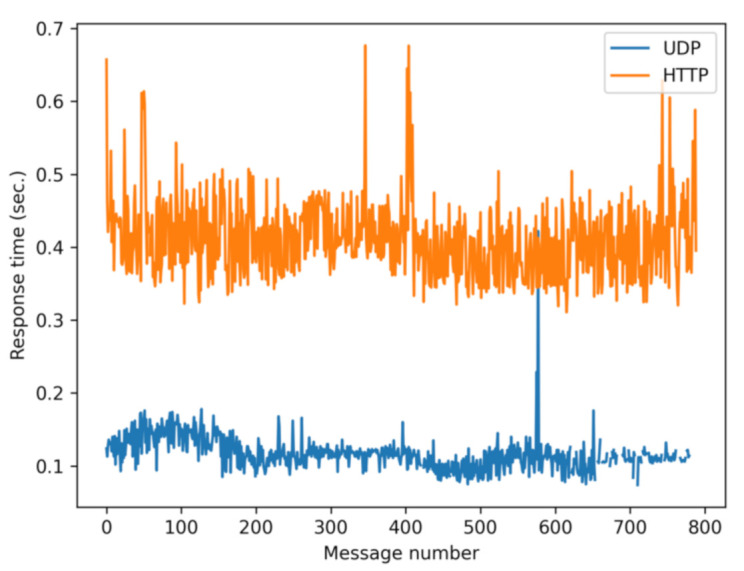
Response time of a trip by bike (20 km/h) comparing the response time with the two versions of the SafeCyclists application.

First of all, to illustrate the difference between the two versions, Figure 4 shows a typical trace of the response time. It can be seen that the UDP version (blue) is on average more than three times faster.

**Figure 5:**
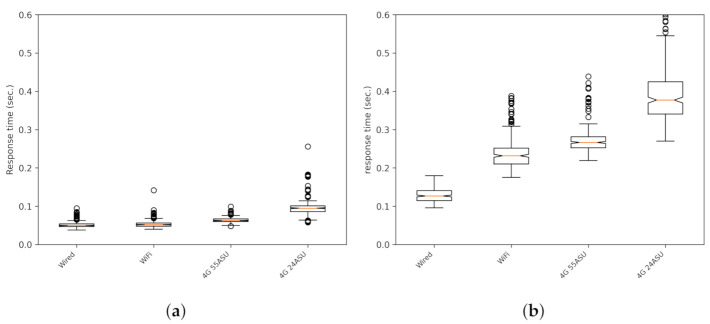
Response times for static clients with either UDP or HTTP and for different types of network connections. In these tests, there were no outdated messages. (**a**) UDP; (**b**) HTTP. ASU, arbitrary strength unit.

**Figure 6:**
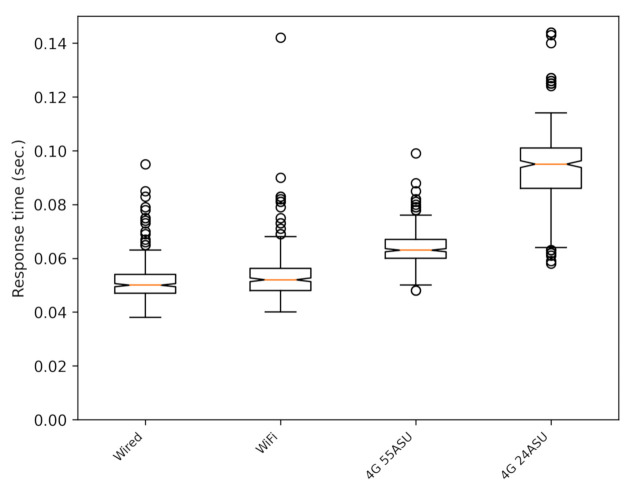
Response times for static clients with the UDP version with different types of network connections. The same results as Figure 5a with an appropriate scale.

In Figure 5a (SafeCyclistsUDP), Figure 5b (SafeCyclistsHTTP), a comparison of both versions is shown as boxplots (In all boxplot figures in the paper, the circles are outliers. These values are bigger than 1.5 times the interquartile range, and they allow for a better representation of the dispersion of the data set. Note that these outliers are not outdated messages, and they are considered correct for our system.). In this case, the tests correspond to static clients, and the experiments correspond to different connection types: wired connection, with a Huawei smartphone connected via WiFi (same Internet connection a the wired case), and the smartphone connected by the cellular 4G LTE telephony data with good (55 ASU) and poor (24 ASU) signal strength levels (the value used to express the signal strength is the arbitrary strength unit (ASU), given that, contrarily to dBm, this unit provides a unified idea of the signal power independently of the cellular phone technology used along a route [26]). It can be seen that the response time is, again, at least three times better in the UDP than in the HTTP version, all with similar levels of lost or outdated response messages (responses arriving after the one second deadline). Both figures have the same scale. Figure 6 shows the same data as Figure 5a, but with an adapted scale.

**Figure 7:**
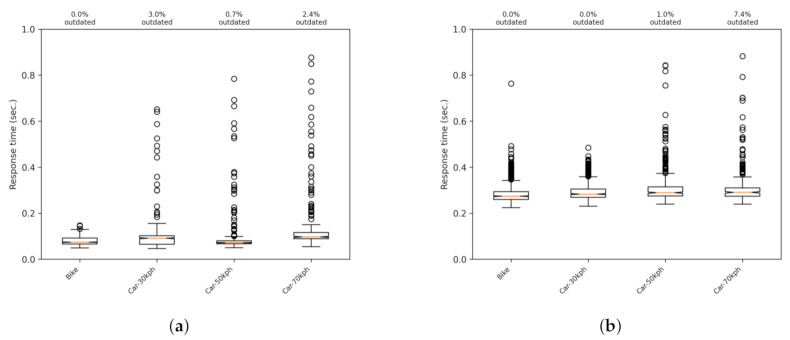
Response times for phones moving at different speeds with the UDP and HTTP communications versions. (**a**) UDP; (**b**) HTTP

**Figure 8:**
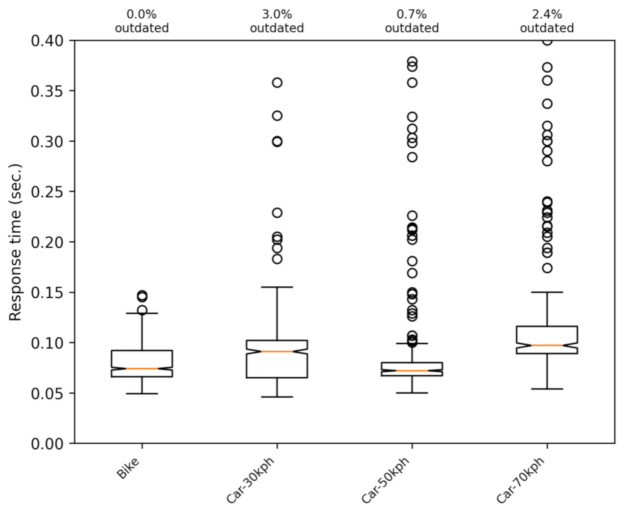
Response times for phones moving at different speeds with the UDP communications version. Same results as Figure 7a with a best suited scale.

We extended the set of tests by introducing mobility to verify the difference between the two communication options. Figure 7a,b shows boxplots when having the phone moving at different speeds, ranging from 15 to 70 km/h in an interurban scenario. The difference between both versions is maintained, and the same order is preserved regarding the percentage of lost or outdated messages. Figure 8 shows the results of the UDP version with a more representative scale.

In view of the results, the SafeCyclistsUDP version is used for the data presented in the rest of this paper as it clearly offers lower response times.

### 5.2. Effect of Signal Strength

The SafeCyclists application deals with mobile clients that should have response times lower than one second. In this scenario, we need to know if there is a strong correlation between the signal strength of the phone’s connection and the response time. To check this relation, we performed many tests, recording the response time, signal strength, and cellular phone technology.

**Figure 9:**
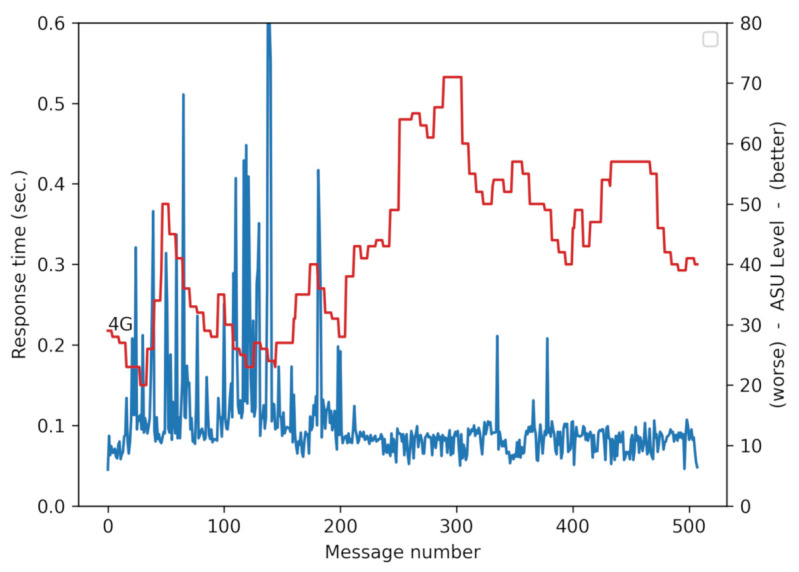
A trace of a trip showing the evolution of signal power (expressed in ASU) and response time.

**Figure 10:**
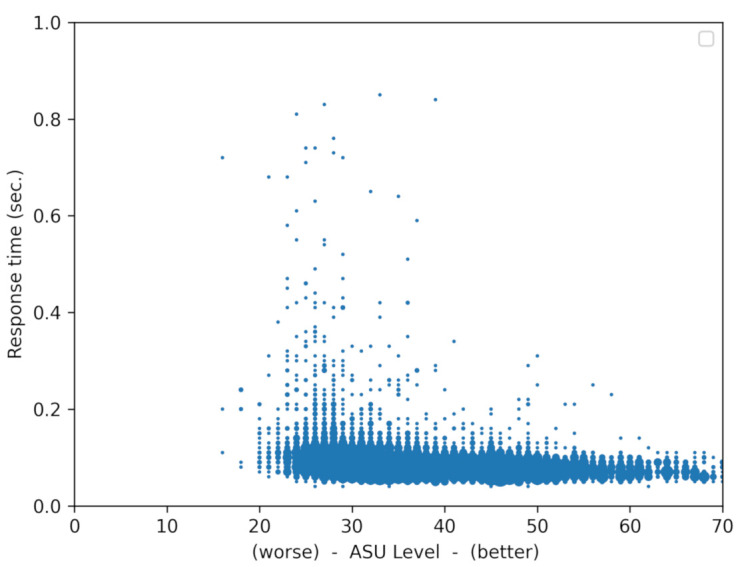
Relation between response time and signal power. The data correspond to the accumulation of several trips (13,098 messages in total).

An example of the behavior of the response time versus signal strength can be seen in Figure 9 and Figure 10.

Figure 9 shows a trace of a suburban car trip with the lines of the signal power and response time over time. It can be seen that, when the signal strength is higher (red line), the response time is reduced (blue line).

Figure 10 was obtained by accumulating the data of trips with different routes and different speeds. This figure represents 13,098 messages with 2015 outdated messages (15.5%), that is messages arriving after the deadline of one second (the percentage of outdated messages provides an idea of the reliability of the system, as if the outdated message is an alarm notification, it would arrive too late to the vehicle). This figure shows that there is a correlation between response time and signal power. The L shape shows a weak correlation between the two variables, given that, with poor signal levels, there is a moderate increase of the response time. When the signal level improves, the values of the response time tend to stabilize.

In any case, we verified that the quality of the signal is not determinant to provide response times below one second and that, if the phone does not lose the connection, there is not a drastic increment in terms of outdated messages.

### 5.3. Impact of the Number of Clients

At the time of writing this paper, the application is not available for general users, because it is still in an experimental stage. Consequently, our server load is limited to a certain amount of real clients. Furthermore, the server part of the system may be improved and optimized through many different techniques. For the tests in this paper, we solely validate the communications part of the system. In any case, we wanted to check the impact of the number of users over the response time.

To load the server, we developed a script to emulate the desired number of clients on different computers. Thus, we can have a real mobile client and test the response time with a different amount of synthetic clients.

The server ran on a virtual machine with two CPUs and 4 Gbytes RAM (and 4 Gbytes of swap) running Debian GNU/Linux 10.7. The server was implemented with Java (openjdk-11.0.9.1). The computer hosting the virtual machine was an eight core Intel Core(TM) i7-8700 CPU 3.20GHz with 24 Gbytes of RAM also running Debian GNU/Linux 10.7.

In the implementation of the server application, each message received by the server is handled by a different thread, which is destroyed after serving the client.

**Figure 11:**
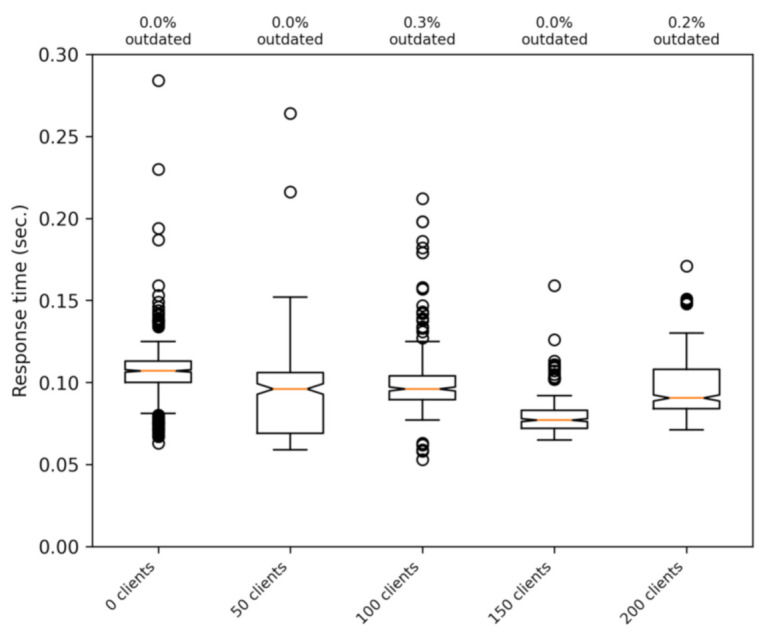
Effect on the response time of a real client of a variable number of concurrent synthetic clients.

Figure 11 shows the response time with a different number of simultaneous clients. In these tests, the real client has a good signal strength (between 25 and 30 ASU), and to discard interference with other phenomena, we used a static client. It can be seen that the response time is not affected by the number of clients until both CPUs of the virtual computer running the server is at 100% of usage. The saturation of the server is reached when having about 250 clients, and this is mainly due to the tasks performed by the PostgreSQL database to handle messages. In the tests shown in the figure (up to 200 clients), there are no outdated messages. This means that the bottleneck is our simple version of the server, and not the communications part of the system.

### 5.4. Battery and Data Consumption

**Figure 12:**
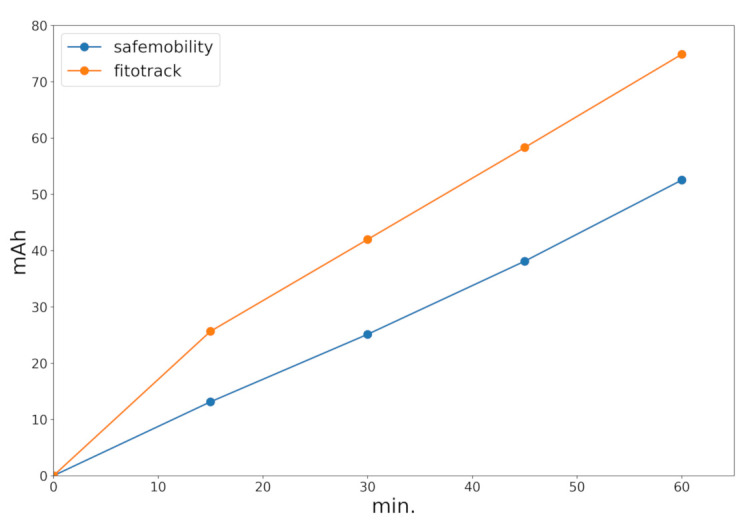
Battery consumption comparison for the Fitotrack and SafeCyclists applications over time. In both cases, the phone screen was turned off.

Another important aspect for a wide acceptance of the application is battery consumption, especially for non-motorized vehicles without a power supply. We compared the application energy consumption against Fitotrack [27], another application that only makes use of GPS. These power consumption values are shown in Figure 12. The data in this figure correspond to one hour using the applications with the screen turned off. As can be seen, the consumption of our SafeCyclists application is slightly less than the Fitotrack application, and we found that battery consumption remained below 2% for a smartphone with a 3000 mAh battery (which is a standard capacity currently).

Repeating the tests with the screen turned on showed that the battery consumption was almost multiplied by three. It must be said that users do not need to see the screen to use the application, unless they also want to see the track of their route on the map.

**Figure 13:**
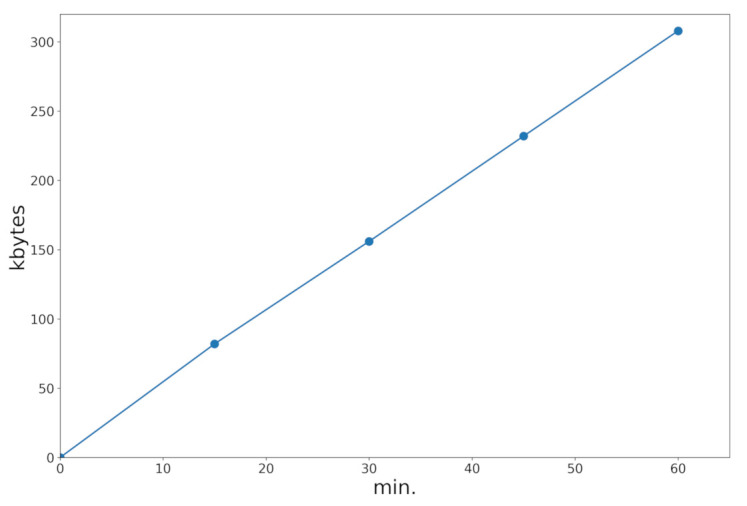
SafeCyclists data consumption over time.

Data consumption is another aspect of interest for users having a limited quota of data. We measured this consumption, finding it to be about 300 kbytes per hour if the application was just used to periodically send the position (see Figure 13). This is a reasonable rate of consumption compared with many common-use applications. If the user wants to see his/her position on a map, then the map tiles could eventually be downloaded as well (about 3 Mbytes an hour on foot). However, in any case, map tiles can be cached, and they are not necessary to use the proposed alarm system.

### 5.5. Impact of the Smartphone Model

**Figure 14:**
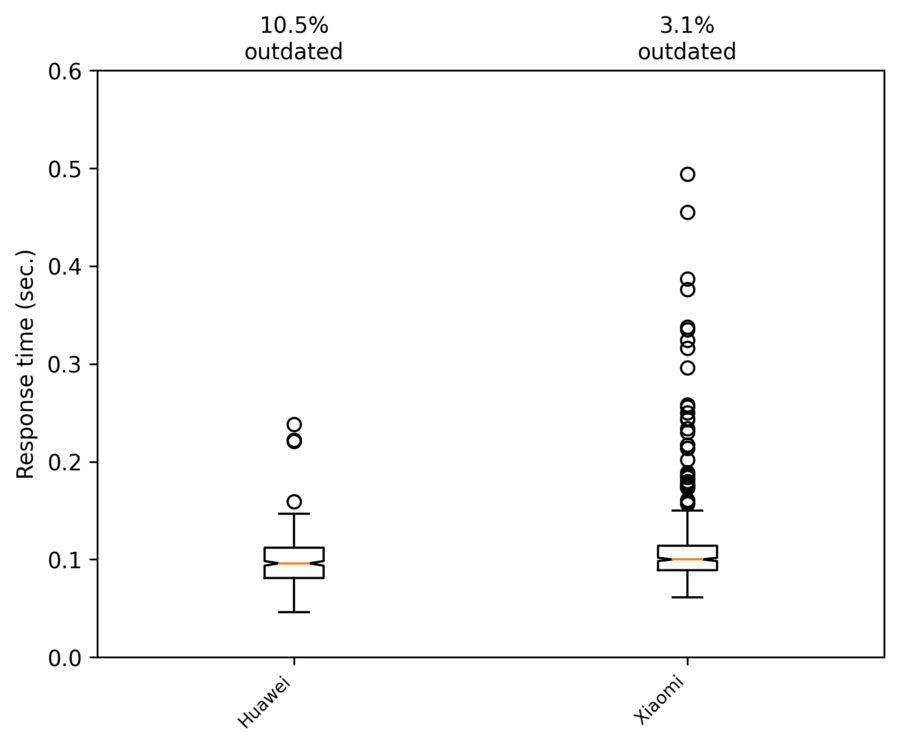
Comparing the two smartphones used in the tests. The data shown correspond to a walking trip.

**Figure 15:**
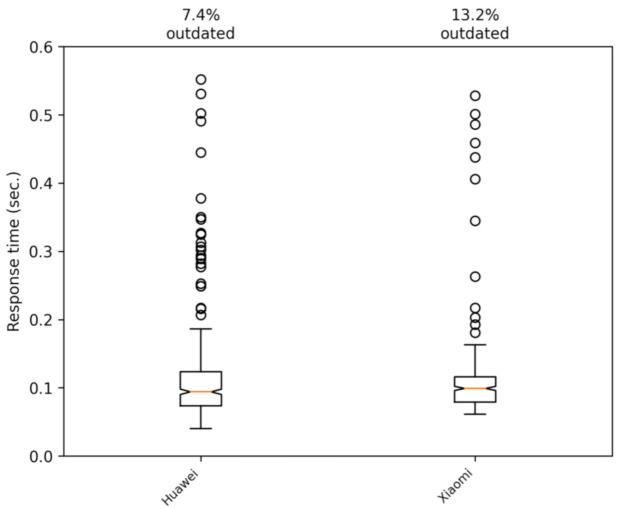
Comparing the two smartphones used in the tests. The data shown correspond to a car trip.

Next, we proceeded to determine if there were significant performance differences when comparing different smartphones. Thus, two mid-range smartphones were used in our tests: a Huawei P30 Lite and a Xiaomi Rednote 9 Pro. The telecommunications operator in both cases was Simyo (https://www.simyo.es, accessed on 15 March 2021), which operates over the mobile infrastructure of the Orange company (https://www.orange.es, accessed on 15 March 2021) in Spain. We performed some tests to verify that the phones models we used in the experiments had no effect on the response time of the application. The tests were carried out on the campus of Jaume I University on foot and by car. The results are shown in Figure 14 and Figure 15. These figures show that the differences between the two phones are mostly insignificant.

## 6. Functional Validation: Checking the Accuracy of Distance Warnings

In this section, our goal is to make a functional validation of our architecture. To this end, we performed actual field experiments by equipping both a bike and a car with our system running on a smartphone on each vehicle.

The system acts as follows: when the server receives a position update, it checks if the sender has pending alarms, i.e., other vehicles closer than 150 m, and it sends this information back to the vehicle.

The computation time to match clients with alarms, if only two clients are using the application, is obviously negligible, but it allows us to check the accuracy of the alarm system mainly by allowing us to determine the cost and delay of the communications.

**Figure 16:**
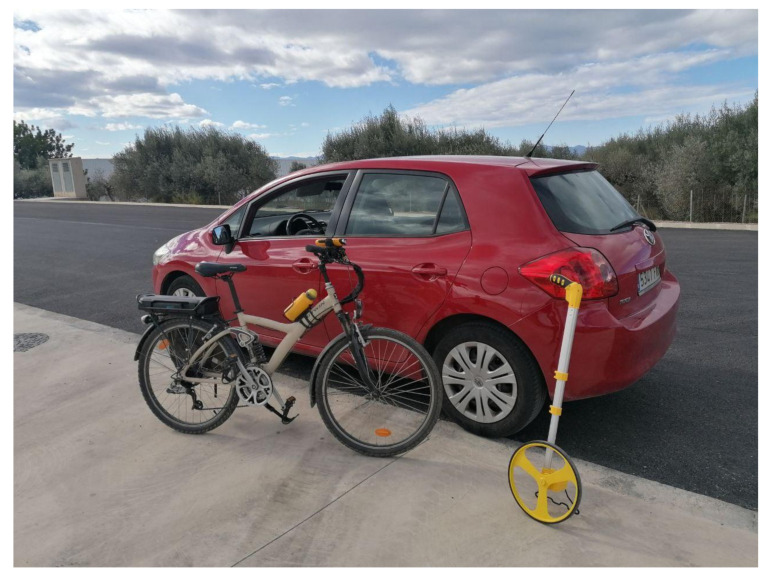
The car, the ebike, and the topometer used in the validation tests.

We performed tests with a car and a bike (Figure 16) in different scenarios. Each case was repeated three times, and the results obtained were the mean and standard deviation, the latter being quite reduced. These tests were performed in an interurban road next to an industrial area.

As a preliminary verification, we conducted tests in a scenario where both vehicles were together (the alarm was on) and the bicycle began to move away at 20 km/h until the alarm went off. The measured distance in these tests was around 159 m, showing that the threshold setting was correctly defined and applied.

**Figure 17:**
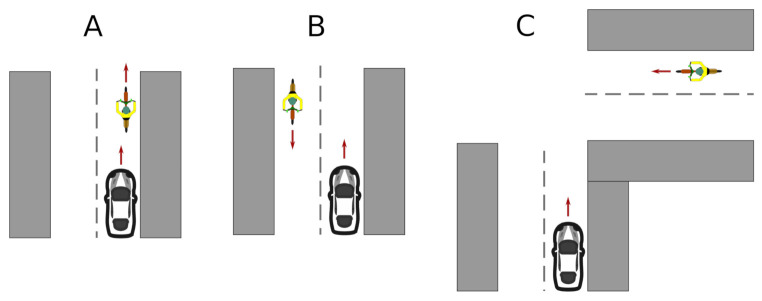
Scenarios to validate the alarm system. (**A**) both vehicles traveling in the same direction. (**B**) vehicles traveling in the opposite direction. (**C**) vehicles meeting at a 90° road crossing.

The remaining scenarios are shown in Figure 17, and they consisted of: both vehicles traveling in the same direction (A), in the opposite direction (B), or meeting at a 90° road crossing (C).

**Figure 18:**
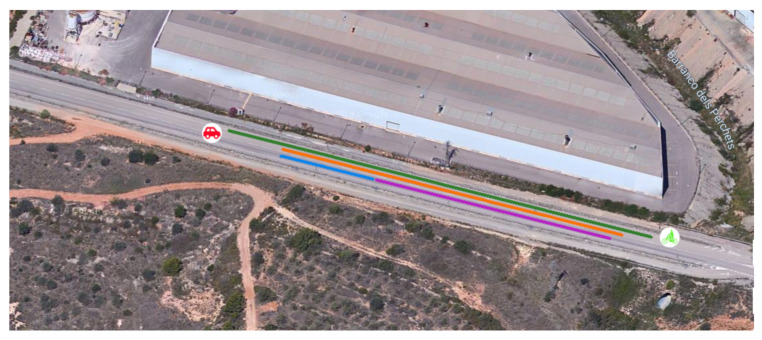
Image with distances for the worst case (car and bicycle in opposite directions with a relative speed of 90 km/h). The green line is the alarm threshold (150 m)l the orange line shows the distance when the alarm pops up (121 m); the blue line is the car breaking distance (31 m); and finally, the purple line is the remaining distance when the two vehicles are stopped (88 m). Note that the reaction time in the experiment is quite reduced because the driver is waiting for the alarm.

Table 1 and Table 2 show the distance at which the warning is shown to the user of the application when both vehicles move in the same direction and in the opposite direction, respectively. In all cases, the bike was moving at 20 km/h, while the car moved at 30, 50, and 70 km/h. In these tables, it can be seen that, when increasing the relative speed, the distance at which the alarm pops up was reduced. Furthermore, the accuracy of the GPS must be taken into account, as it is known to introduce an uncertainty of about 5 m even when it is working under the best case scenario [12]. In any case, the distance needed to stop the car before reaching the bicycle was found to be enough in all test cases, as can be seen in Figure 18.

In the case of the perpendicular crossroad, the tests were conducted under an inverse assumption: starting at the intersection and moving at a relative angle of 90°, because this way, it was easier to guarantee coincidence at the intersection itself. Thus, the alarm was initially on, and we measured the distance traveled by each vehicle until the alarm went off. These tests were conducted with the bike moving at 20 km/h and the car at 30 km/h. We did not test other speeds, keeping in mind that the car should reduce its speed when arriving at an intersection. The results were 125 and 77 m for the car and the bike, respectively. In these cases, the proximity distance was measured diagonally, resulting in 147 m.

## 7. Conclusions and Future Work

There is an urgent need to reduce our carbon footprint and to improve everyone’s quality of living. The use of lightweight vehicles in our usual commuting or in sports practices is proven to be effective to reduce noise and air pollution and to ameliorate the general health level. However, traffic safety is a major concern in this behavioral change. In this paper, we prove that a traffic alarm system can be directly adopted based on the ubiquity of the current cellular phone infrastructure and smartphones.

In effect, in the present work, we test our solution, where the system communications relied on a 4G cellular network. We obtain response times of about 0.1 s, and we test it in different real conditions. Experiments show that alarms are triggered when expected, with only a reduced amount of messages arriving after the deadline defined (one second). These low time offsets enable, for instance, warning drivers about the presence of bicycles closer than 150 m (or at a distance defined according to the relative speed of the vehicles and the weather); the relative position of these nearby vehicles or other data could also be included. This way, the driver has time to pay more attention to a dangerous event or to stop the car before a collision. This system does not depend on the line of sight, nor on other obstacles or vehicles between them.

However, the system is not designed as a critical collision avoidance system. In this case, the proximity to trigger the alarm should be reduced to distances of a few tens of meters, and then, the alarm could be set off too late. On the other hand, if the proximity distance is incremented, then it can produce many false positives, and users could loose their confidence.

Once having proved that the system is feasible, and better knowledge of its limits, other studies and improvements can take place. For instance, different technologies for the asynchronous alarm notification from the server to clients can be tested. The instant for sending the position could be based on the traveled distance rather than on a fixed time period. A richer dataset could be included in the alarms, such as the direction and speed of the vehicles triggering the event. Alarms could be selected more accurately, for instance by taking into account the direction of vehicles or the road width. Finally, several algorithms and improvements can be tested in the server part to increase its efficiency and capacity.

## Figures and Tables

**Table 1:** Distance at which the alarm is displayed when the vehicles moves in the same direction.

Same Direction	Warning Distance (m)	Deviation (m)	Relative Speed (km/h)
car @ 30 km/h	167	5.5	10
car @ 50 km/h	152	5.2	30
car @ 70 km/h	141	3.7	50

**Table 2:** Distance at which the alarm is displayed when the vehicles moves in the opposite directions.

Opposite Direction	Warning Distance (m)	Deviation (m)	Relative Speed (km/h)
car @ 30 km/h	142	5.0	50
car @ 50 km/h	129	2.5	70
car @ 70 km/h	121	10.7	90
